# Cross-sectional study on the association between the fibrosis-4 index and co-occurring myocardial infarction in Chinese patients with type 2 diabetes mellitus

**DOI:** 10.3389/fendo.2025.1551472

**Published:** 2025-03-12

**Authors:** Ziyi Sun, Jin Zhang, Jinlong Duan, Qingqing Wang, Zhangjun Yun, Jianguo Lin, Yuhan Yang, WenXi Zuo, Zeqi Wang, Xingjiang Xiong, Kuiwu Yao

**Affiliations:** ^1^ Department of Cardiology, Guang’anmen Hospital, China Academy of Chinese Medical Sciences, Beijing, China; ^2^ Graduate School, Beijing University of Chinese Medicine, Beijing, China; ^3^ Department of Andrology, Guang’anmen Hospital, China Academy of Chinese Medical Sciences, Beijing, China; ^4^ Department of Internal Medicine, Eye Hospital China Academy of Chinese Medical Sciences, Beijing, China; ^5^ Department of Oncology and Hematology, Dongzhimen Hospital, Beijing University of Chinese Medicine, Beijing, China; ^6^ School of Traditional Chinese Medicine, Beijing University of Chinese Medicine, Beijing, China; ^7^ Academic Administration Office, China Academy of Chinese Medical Sciences, Beijing, China

**Keywords:** type 2 diabetes mellitus, myocardial infarction, FIB-4, biomarker, cross-sectional study

## Abstract

**Background:**

Previous studies indicated that the Fibrosis-4 Index (FIB-4), an evaluation metric for liver fibrosis, is associated with adverse outcomes in coronary artery disease. However, the correlation between FIB-4 and myocardial infarction (MI) in Chinese patients with Type 2 Diabetes Mellitus (T2DM) has not been well-defined. Thus, this study aims to elucidate the association between FIB-4 and MI in Chinese T2DM patients.

**Methods:**

Cross-sectional data were collected from T2DM patients at two hospitals in China, designated as the discovery and validation centers. The exposure variable, FIB-4 index, was derived from patient age, aspartate aminotransferase (AST), alanine aminotransferase (ALT), and platelet count. This index was stratified into four distinct clusters via k-means clustering analysis. The primary outcome was defined as the incidence of co-occurring MI. Logistic and restricted cubic spline regression was conducted to assess the association between the FIB-4 index and MI in Chinese T2DM patients.

**Results:**

In the discovery phase, data were analyzed from 2,980 T2DM patients, including 1,114 females (37.38%), with 58 years average age (SD: 10.4). Among them, 190 were also MI patients. Based on the fully adjusted logistic regression analysis, the odds ratio (OR) for the second cluster was 1.00 (95% CI, 0.60-1.40); for the third cluster, it was 1.94 (95% CI, 1.32-2.57), and for the poorest controlled cluster it was 16.18 (95% CI, 14.97-17.39) in comparison to the best-controlled cluster of FIB-4. Restricted cubic spline regression revealed a linear relationship between the FIB-4 index and MI risk. Subgroup analysis demonstrated that this association was significant in elderly adults, females with high BMI, and those with comorbidities such as hypertension, coronary artery disease, and chronic heart failure. These findings yield consistent results in the validation set (n = 224).

**Conclusions:**

Among Chinese patients with T2DM, elevated FIB-4 levels have been independently associated with MI, particularly among females and individuals with concomitant hypertension. Consequently, the FIB-4 index is anticipated to serve as a promising tool for early detection and risk stratification in this population.

## Introduction

Type 2 diabetes mellitus (T2DM) is a chronic metabolic disorder characterized by hyperglycemia, with its prevalence continuously rising globally, particularly in China (1, 2). T2DM not only leads to microvascular complications, such as diabetic nephropathy and retinopathy, but also significantly increases the risk of macrovascular events, including atherosclerotic cardiovascular disease (ASCVD) and myocardial infarction (MI) (3). Moreover, poor glycemic control is directly associated with both short- and long-term adverse outcomes following MI (4, 5). Although new T2DM therapies, including sodium-glucose cotransporter-2 (SGLT-2) inhibitors and glucagon-like peptide-1 (GLP-1) receptor agonists, have demonstrated cardiovascular benefits (6, 7), it is still crucial to identify and manage T2DM patients who are at risk of developing MI at an early stage.

There is a well-established pathophysiological link between T2DM and liver disease, particularly metabolic dysfunction-associated fatty liver disease (MASLD). Approximately 55.5% of T2DM patients also present with MASLD, and 37.3% of these progress to metabolic dysfunction-associated steatohepatitis (MASH) (8). Insulin resistance, chronic inflammation, and lipid metabolism disturbances collectively contribute to the development of liver fibrosis (9). Liver fibrosis is not only a critical stage in liver disease progression but may also exacerbate cardiovascular disease (CVD) risk through mechanisms such as systemic inflammation, oxidative stress, and endothelial dysfunction (10). Studies indicate that the risk of CVD is nearly doubled in T2DM patients compared to those with isolated MASLD (OR=1.92), with a significant increase in cardiovascular mortality (11, 12). Notably, the presence of advanced liver fibrosis further elevates CVD mortality by 64% (13). Therefore, non-invasive markers of liver fibrosis may provide novel insights into cardiovascular risk stratification in T2DM patients.

The fibrosis-4 (FIB-4) index, a non-invasive scoring system based on age, aspartate aminotransferase (AST), alanine aminotransferase (ALT), and platelet count (PLT), offers a simple method for assessing liver fibrosis (14). FIB-4 has demonstrated similar or superior performance to a range of fibrosis biomarkers (15, 16), and it is consistently recommended in international guidelines as part of the first-line assessment for MASLD and T2DM (17, 18). Recently, increasing attention has been given to the role of FIB-4 in predicting CVD progression and prognosis. Research shows that FIB-4 is significantly associated with adverse outcomes in conditions such as heart failure (HF), stroke, and coronary heart disease (CHD) (19–21). However, the relationship between FIB-4 and MI in Chinese T2DM patients has not been conclusively established. Previous studies, such as that by Song et al. (22), evaluated the impact of FIB-4 on cardiovascular events in acute coronary syndrome (ACS) patients, with and without T2DM. The results indicated that intermediate to high FIB-4 scores (>1.45) were significantly associated with an increased risk of major adverse cardiac events (MACE), MI, and CVD mortality in ACS patients. Further subgroup analysis revealed that this association remained significant in ACS patients with T2DM. Another study by Guan et al. (23), utilizing data from the National Health and Nutrition Examination Survey (NHANES), showed that an increase of one standard deviation in FIB-4 was associated with higher risks of all-cause mortality (HR, 95% CI 1.24, 1.17–1.32) and CVD mortality (HR, 95% CI 1.17, 1.04–1.31) in T2DM patients, with the association being particularly pronounced in those with MI (OR, 95% CI 1.25, 1.05–1.47). Nevertheless, several issues remain to be addressed, including the potential impact of chronic liver and biliary diseases on FIB-4, which has not been adequately corrected for in previous studies. Furthermore, the applicability of FIB-4 risk thresholds in Chinese T2DM patients warrants further investigation. Lastly, the relationship between FIB-4 and MI in T2DM patients requires clarification in specific subgroups.

In light of the above, this study aims to explore the association between the FIB-4 index and MI in T2DM patients using cross-sectional data from two hospitals in China. By adjusting for potential confounders and analyzing subgroup differences, we seek to provide new evidence for cardiovascular risk stratification in T2DM patients and lay the groundwork for future clinical intervention strategies.

## Materials and methods

### Data sources and study population

**Figure 1:**
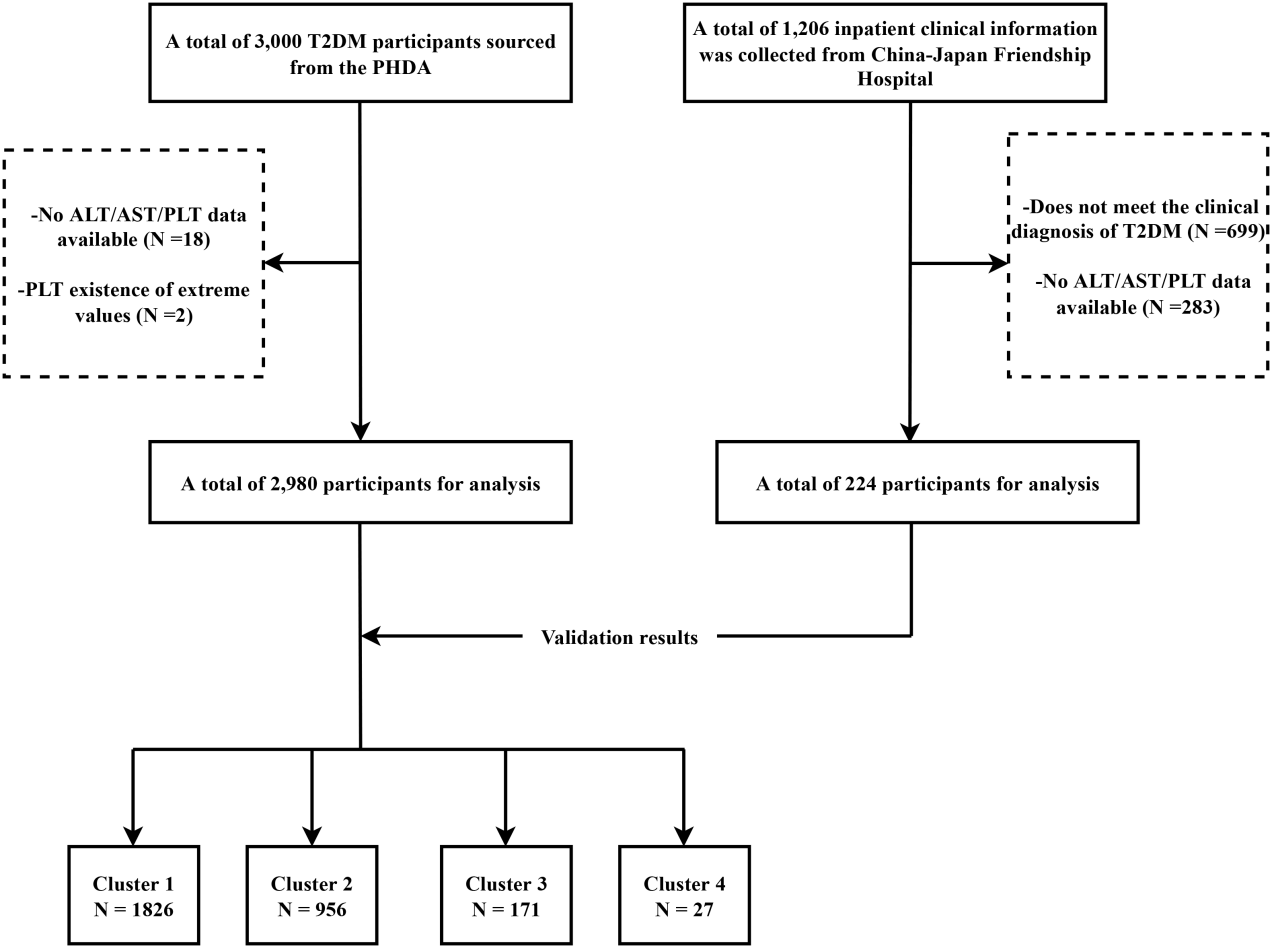
Flowchart of the study design. T2DM, Type 2 Diabetes Mellitus; PHDA, Population Health Data Archive; ALT, alanine aminotransferase; AST, aspartate aminotransferase; PLT, platelet count.

This study collected comprehensive data from two hospitals. The primary analysis, referred to as the discovery phase, used data from the Population Health Data Archive (PHDA), managed by the National Center for Medical Intelligence, China (https://www.ncmi.cn) (24), provided by the Chinese People’s Liberation Army General Hospital. The dataset comprised demographic details, medical history, and laboratory measurements for 3,000 T2DM patients. Participants without data on ALT, AST, or PLT (n = 18) and those with PLT values < 10 (n=2) were excluded, resulting in a final 2,980 participants. Data for the validation phase were collected from hospitalized patients at the Department of Integrative Cardiology, China-Japan Friendship Hospital, between July 2017 and January 2023. Data were collected via the hospital’s electronic medical record system. Inclusion criteria were defined as follows (1): participants aged over 18 years; (2) a discharge diagnosis that concurrently included T2DM (ICD-10 code: E11 or its subcategories) and MI (ICD-10 codes: I21, I22, or I25.2); and (3) the availability of the primary observational variables required for this study. Participants without data for ALT, AST, or PLT (n=283) were excluded, leaving 224 patients for the validation analysis. A flowchart of the screening of the study population is illustrated in **Figure 1**.

All PHDA data had undergone prior ethical review by the original institutions, ensuring compliance with regulations. As anonymized, publicly available data, separate trial registration was not required. The data used in the validation phase were obtained from a medical records system and were anonymized. Ethical review exemption was granted by the Ethics Committee of China-Japan Friendship Hospital. This study was conducted in accordance with the Declaration of Helsinki and relevant guidelines.

### Data collection

The covariates were selected based on previous studies, focusing on the main factors that could substantially impact evaluating the association between FIB-4 and comorbid MI in T2DM patients (22, 25, 26). In the discovery phase, the following data were collected: (i) Demographic details: sex, age, and marital status; (ii) Physical measurements: systolic blood pressure (SBP), diastolic blood pressure (DBP), and body mass index (BMI); (iii) Comorbidities: MI, CHD, AS, hypertension, HF, dyslipidemia, diabetic retinopathy, chronic liver disease, biliary tract disease, renal impairment, etc.; (iv) Laboratory tests: glycosylated hemoglobin A1c (HbA1c), fasting blood glucose (FBG), triglyceride (TG), total cholesterol (TC), high-density lipoprotein cholesterol (HDL-c), low-density lipoprotein cholesterol (LDL-c), ALT, AST, PLT, etc. Due to data storage limitations, detailed medical histories were not obtained in the validation phase; only hypertension, CHD, and MI information were collected. Moreover, data on left ventricular ejection fraction (LVEF), N-terminal pro-b-type natriuretic peptide (NTpro-BNP), hemoglobin (Hb), and serum creatinine (Cr) were collected for further adjustments.

### Calculation of FIB-4

In this study, FIB-4 was designated as the exposure variable. The following formula was used to calculate the FIB-4 index:

FIB−4 = [(Age (years) × AST (U/L))/(PLT (10^9^/L) × ALT^1/2^(U/L)] (14).

### Outcome assessment

The primary outcome measured in this study was the comorbidity of MI. Data were obtained from hospital records for the discovery and validation phases, ensuring accurate detection and assessment of MI events.

### Missing variables

Variables containing > 30% missing data were removed. The distribution of missing variables within this study is presented in **Supplementary Figure S1**. Multiple imputation was used to maintain the largest possible sample size and address missing data despite the small percentage of missing data. The study also used the ‘mice’ package in R for multiple imputations, with at least five imputed datasets and fifty iterations per dataset.

### Statistical analysis

**Figure 2:**
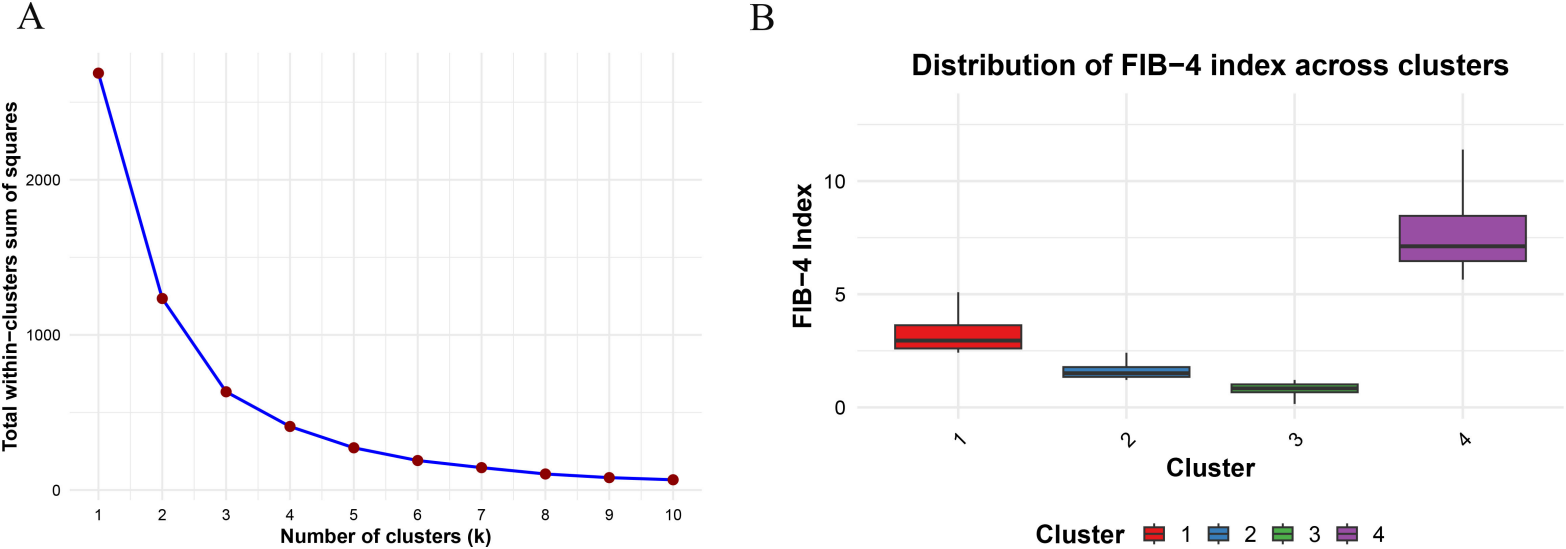
**(A)** Clustering of Fibrosis-4 index by K-means clustering; **(B)** Distribution of Fibrosis-4 index across different clusters.

An iterative optimization method, K-means clustering, minimizes the distances between data points and their centroids to identify clusters. It is known for its scalability and simplicity (27, 28). Four clusters were identified as the optimal number in this study. As a result, the study population (n = 2980) was divided into four groups based on their FIB-4 index, indicating varying control levels from optimal to poor. For the first cluster, FIB-4 ranged from 0.150 to 1.211, suggesting the best control of FIB-4 levels; for the second cluster, FIB-4 ranged from 1.212 to 2.412, indicating better control; for the third cluster, FIB-4 ranged from 2.413 to 5.404, representing poorer control; and for the fourth cluster, FIB-4 ranged from 5.645 to 13.219, depicting the poorest control (**Figure 2**).

Basic characteristics were described as mean ± SD for normally distributed data, medians with interquartile ranges for non-normally distributed data, and percentages for categorical data. When comparing the means of two groups, an independent sample t-test is used if the data follow a normal distribution with equal variances. The Wilcoxon rank-sum test is applied for non-normally distributed data. For comparisons involving continuous variables across multiple groups, one-way ANOVA is performed if the data are normally distributed with equal variances, followed by pairwise comparisons using the Tukey HSD method. If the data are non-normally distributed or variances are unequal, the Kruskal-Wallis rank-sum test is utilized, with pairwise comparisons adjusted by the Dunn-Bonferroni method. Chi-square tests or Fisher’s exact probability test are used for categorical variable comparisons.

Univariate and multivariate logistic regression models were used to assess the relationship between FIB-4 levels and the co-occurrence of MI, providing OR with 95% confidence intervals (CI). Model 1 adjusted for age and gender. Model 2 was further adjusted for age, gender, CHD, hypertension, HF, diabetic retinopathy, hyperlipidemia, AS, stroke, carotid occlusion, and arrhythmias. Model 3 expanded upon Model 2 by further adjusting for fatty liver, cirrhosis, other chronic liver diseases, biliary diseases, pancreatic diseases, renal dysfunctions, gastrointestinal tumors, hematologic disorders, BMI, HbA1c, FBG, TG, HDL-c, and Hb. Before regression analysis, variables with a variance inflation factor (VIF) >5 were excluded to prevent multicollinearity issues (**Supplementary Table S1**). Multivariable-adjusted restricted cubic spline (RCS) and logistic regression analyses were conducted using four knots at the 5th, 35th, 65th, and 95th percentiles to explore the nonlinear and dose-response relationships between FIB-4 and MI (29). Trend tests evaluated the association between increasing FIB-4 levels and comorbid MI. Moreover, subgroup analysis and interaction (likelihood ratio) tests were performed to identify potential biases. Participants were categorized into subgroups according to age (< 65 and ≥ 65 years), sex, BMI (< 23.9 and ≥ 23.9), and the presence of hypertension, CHD, HF, hyperlipidemia, AS, stroke, diabetic retinopathy, fatty liver, and other chronic liver diseases. All statistical analyses were performed via R software v4.3.1 (http://www.R-project.org/) and IBM SPSS Statistics v27.0. A two-tailed *p*-value < 0.05 was considered statistically significant.

### Sensitivity analysis

A series of sensitivity analyses were performed to validate the reliability and stability of the study findings. First, logistic regression models were adjusted within the five imputed datasets to minimize the potential differences among the imputed data on the primary outcome. The association between previously defined risk thresholds of FIB-4 (low risk < 1.3; intermediate risk 1.3 to 2.67; high risk >2.67) and co-occurring MI was also investigated (18). Further, an imbalance in patient distribution across clusters was observed after k-means clustering, particularly in those with higher FIB-4 values (Clusters 3 and 4). This uneven distribution may influence the stability and interpretability of the statistical results. To ensure the reliability of the findings, the stability of the results was further tested by categorizing based on the quintiles of FIB-4 and implementing 1:1 propensity score matching (nearest neighbor matching). The association between FIB-4 and MI was re-examined in further validation datasets (n = 224). Additionally, the receiver operating characteristic (ROC) curve analysis was conducted to assess the discriminative ability of the FIB-4 index for MI. The Youden index and optimal cutoff value were calculated to determine the utility of FIB-4 in identifying MI risk in this population.

## Results

### Baseline characteristics of participants

In the study’s discovery phase, 2,980 patients with T2DM were included, with an average age of 58 ± 10.4 years. Among these patients, 1,114 (37.38%) were females, and 190 had comorbid MI. Basic information for the validation data is listed in **Supplementary Table S2**.

The basic characteristics of the participants are summarized in **Table 1**. According to the classification results from K-means clustering, participants in Cluster 4, compared to those in Cluster 1 (with the lowest FIB-4 values), were older, had lower blood pressure, higher BMI, and prevalence of MI, CHD, AS, HF, chronic liver diseases, liver cirrhosis, and gastrointestinal tumors. They also showed lower rates of hyperlipidemia and fatty liver disease. Biochemically, Cluster 4 individuals had higher ALT, AST, and PLT levels but lower HbA1c, TC, TG, HDL-c, LDL-c and Hb levels, with all differences being statistically significant (*p* < 0.05). Moreover, baseline characteristics before and after imputation, categorized by the co-occurrence of MI, are provided in **Supplementary Tables S3**, **S4**.

**Table 1:** Baseline characteristics of individuals classified by Fibrosis-4 index clustering.

Characteristics	Cluster1	Cluster2	Cluster 3	Cluster 4	P-value
N	1826	956	171	27	
Gender (Female)	1166 (63.9%)	582 (60.9%)	102 (59.6%)	16 (59.3%)	0.361
Age, years	54.0 ± 10.4	63.4 ± 9.3	66.4 ± 10.6	61.1 ± 11.6	<0.001
Marital status (Married)	1787 (97.9%)	935 (97.8%)	169 (98.8%)	26 (96.3%)	0.776
SBP, mmHg	138.2 ± 20.5	139.6 ± 21.3	141.6 ± 24.4	121.2 ± 15.5	<0.001
DBP, mmHg	81.7 ± 12.0	78.9 ± 11.5	77.3 ± 12.8	72.9 ± 9.9	<0.001
BMI, kg/m^2^	26.6 ± 3.8	26.1 ± 3.8	25.8 ± 4.7	27.8 ± 8.0	<0.001
MI	91 (5.0%)	63 (6.6%)	26 (15.2%)	10 (37.0%)	<0.001
CHD	536 (29.4%)	359 (37.6%)	75 (43.9%)	12 (44.4%)	<0.001
Hypertension	1231 (67.4%)	670 (70.1%)	120 (70.2%)	15 (55.6%)	0.224
Dyslipidemia	428 (23.4%)	195 (20.4%)	28 (16.4%)	2 (7.4%)	0.017
Diabetic retinopathy	946 (51.8%)	474 (49.6%)	61 (35.7%)	7 (25.9%)	<0.001
Arteriosclerosis	888 (48.6%)	541 (56.6%)	93 (54.4%)	16 (59.3%)	0.001
CHF	98 (5.4%)	74 (7.7%)	33 (19.3%)	6 (22.0%)	<0.001
Stroke	122 (6.7%)	83 (8.7%)	17 (9.9%)	1 (3.7%)	0.125
Carotid artery stenosis	78 (4.3%)	46 (4.8%)	5 (2.9%)	0 (0.0%)	0.459
Fatty liver disease	649 (35.5%)	252 (26.4%)	31 (18.1%)	0 (0.0%)	<0.001
Other chronic liver diseases	198 (10.8%)	157 (16.4%)	46 (26.9%)	5 (18.5%)	<0.001
Cirrhosis	6 (0.3%)	13 (1.4%)	20 (11.7%)	8 (29.6%)	<0.001
Pancreatic Diseases	29 (1.6%)	14 (1.5%)	5 (2.9%)	0 (0.0%)	0.487
Biliary tract diseases	243 (13.3%)	143 (15.0%)	35 (20.5%)	4 (14.8%)	0.068
Digestive tumors	66 (3.6%)	65 (6.8%)	19 (11.1%)	3 (11.1%)	<0.001
Renal failure	104 (5.7%)	58 (6.1%)	17 (9.9%)	1 (3.7%)	0.156
LEAD	285 (15.6%)	161 (16.8%)	24 (14.0%)	3 (11.1%)	0.646
ALT, U/L	18.0 (12.7, 26.9)	17.4 (12.4, 26.6)	22.9 (15.6, 40.6)	27.3 (15.3, 44.6)	<0.001
AST, U/L	15.1 (12.4, 18.9)	17.9 (14.5, 23.7)	22.9 (15.6, 40.6)	27.3 (15.3, 44.6)	<0.001
PLT, 10^^9^/L	246.3 ± 70.5	182.0 ± 41.2	141.3 ± 52.9	112.9 ± 70.0	<0.001
Hb, g/L	131.76 ± 23.11	133.32 ± 22.48	130.40 ± 22.33	118.25 ± 30.27	<0.001
FIB-4	0.8 ± 0.2	1.6 ± 0.3	3.2 ± 0.8	7.8 ± 2.1	<0.001
FBG, mmol/L	8.5 ± 3.8	8.2 ± 4.0	8.6 ± 4.0	9.6 ± 4.3	0.087
HbA1c, %	7.9 ± 1.7	7.7 ± 1.7	7.5 ± 1.6	7.6 ± 2.1	0.003
TC, mmol/L	4.7 ± 1.5	4.5 ± 1.3	4.3 ± 1.5	3.9 ± 1.8	<0.001
TG, mmol/L	1.7 (1.2, 2.5)	1.4 (1.0, 2.1)	1.4 (0.9, 2.0)	1.3 (0.8, 1.7)	<0.001
HDL-c, mmol/L	1.1 ± 0.3	1.1 ± 0.3	1.1 ± 0.4	1.0 ± 0.4	<0.001
LDL-c, mmol/L	2.9 ± 1.2	2.8 ± 1.1	2.6 ± 1.1	2.1 ± 1.3	<0.001

SBP, systolic blood pressure; DBP, diastolic blood pressure; BMI, body mass index; CHD, coronary heart disease; CHF, chronic heart failure; LEAD, Atherosclerotic lesions of the lower limbs; ALT, alanine aminotransferase; AST, aspartate aminotransferase; PLT, platelet count; Hb, hemoglobin; FIB-4, Fibrosis-4 index; FBG, fasting blood glucose; HbA1c, glycosylated hemoglobin A1c; TC, total cholesterol; TG, triglyceride; HDL‐c, high‐density lipoprotein cholesterol; LDL-c, low-density lipoprotein cholesterol.

### Odds ratio for co-occurring MI

**Figure 3:**
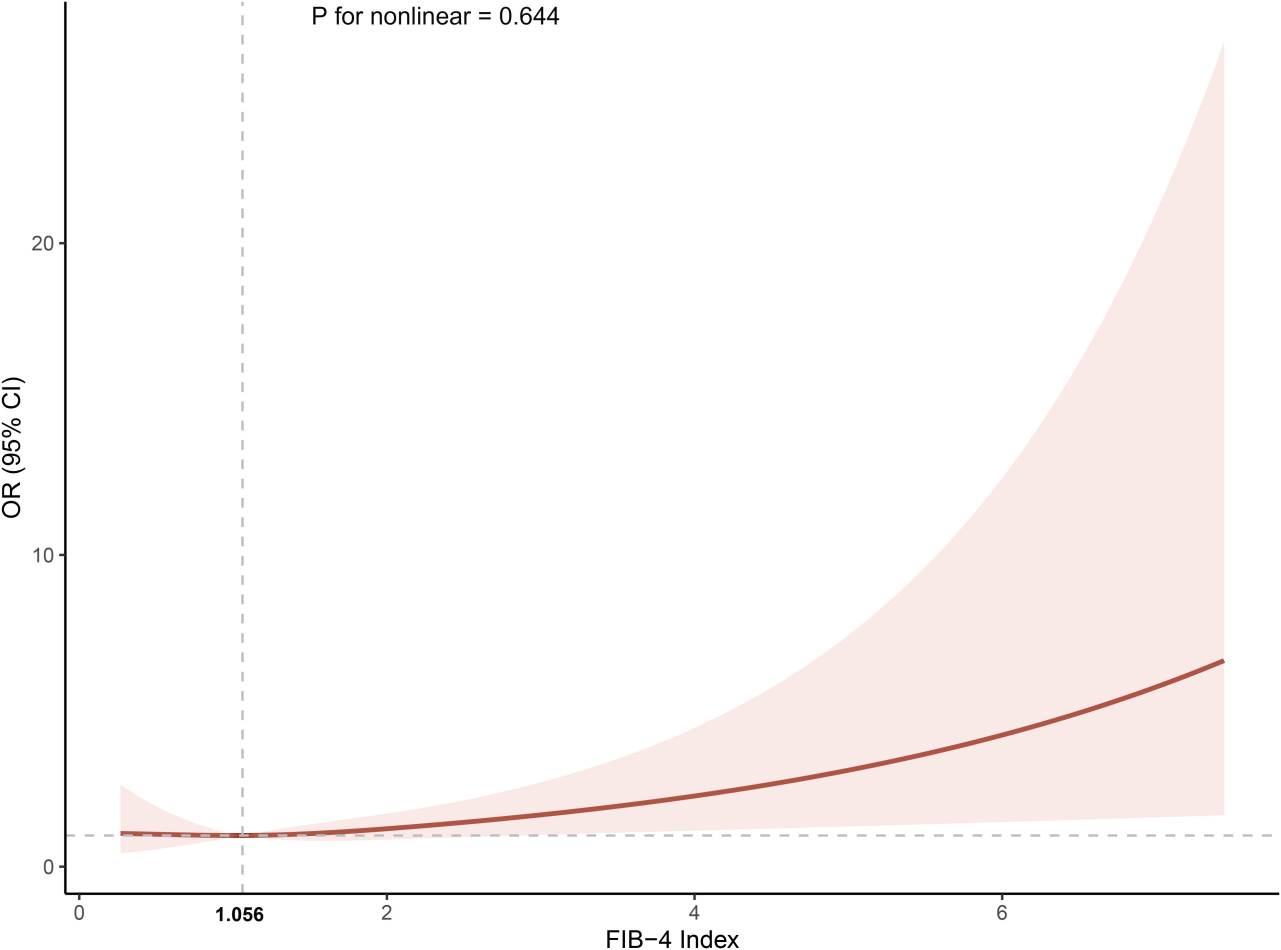
The association between the cumulative Fibrosis-4 index and the incidence of myocardial infarction. The model was adjusted for age, gender, coronary heart disease, hypertension, heart failure, diabetic retinopathy, hyperlipidemia,atherosclerosis, stroke, carotid occlusion, arrhythmias, fatty liver, liver cirrhosis, other chronic liver diseases, biliary diseases, pancreatic diseases, renal impairment, gastrointestinal tumors, hematologic disorders, body mass index, glycosylated hemoglobin A1c, fasting blood glucose, triglyceride, high‐density lipoprotein cholesterol and hemoglobin. OR, odds ratio; CI, confidence interval.

**Table 2** defines the associations between FIB-4 and the probability of MI co-occurrence in different clusters. Three models were constructed based on univariate regression analysis, each representing different adjustment levels for confounding variables. After adjusting for multiple covariates, the fully adjusted logistic regression model indicated that higher levels of FIB-4 (Clusters 3 and 4) were independently associated with the comorbidity of MI in patients with T2DM compared to Cluster 1 (OR, 95% CI: 1.944, 1.323-2.566, Cluster 3; OR, 95% CI: 16.181, 14.972-17.390, Cluster 4). Trend tests indicated a progressive increase in the risk of comorbidity of MI with higher cluster grading. Moreover, when FIB-4 was analyzed as a continuous variable, each SD increase in FIB-4 was associated with a higher risk of comorbidity MI (OR, 95% CI: 1.318, 1.183-1.453). The relationship between FIB-4 and co-occurring CHD was also investigated. Initial findings suggested that FIB-4 levels were correlated with a higher OR of co-occurring CHD (**Supplementary Table S5**). However, this association was rendered non-significant after accounting for potential confounders. Based on this, FIB-4 is more specific to the co-occurrence of MI than CHD in patients with T2DM. The fully adjusted RCS regression model demonstrated a positive linear relationship between FIB-4 and the co-occurrence of MI (overall *p* = 0.037, nonlinearity *p* = 0.644). The predicted OR was approximately 1 when the FIB-4 value was 1.06, as illustrated in **Figure 3**. This suggests that the risk of comorbid MI progressively increases when the FIB-4 value > 1.06.

**Table 2:** Association between Fibrosis-4 index and myocardial infarction.

Fibrosis-4 index	Clusters					Continuous
	Cluster 1	Cluster 2	Cluster 3	Cluster 4	P for trend	Per 1 SD increase
Median	0.84	1.51	2.95	7.12	–	–
Cases, n (%)	91 (5.0)	63 (6.6)	26 (15.2)	10 (37.0)	–	–
Crude, OR (95%CI)	Reference	1.345 (0.966-1.873)	3.419 (2.142-5.457)	11.215 (4.994-25.187)	<0.001	1.405 (1.271-1.554)
Model 1, OR (95%CI)	Reference	0.861 (0.601-1.232)	1.872 (1.234-3.119)	8.766 (3.779-20.333)	<0.001	1.299 (1.169-1.443)
Model 2, OR (95%CI)	Reference	0.931 (0.631-1.373)	1.969 (1.101-3.522)	13.279 (4.285-41.154)	<0.001	1.309 (1.146-1.495)
Model 3, OR (95%CI)	Reference	0.995 (0.596-1.395)	1.944 (1.323-2.566)	16.181 (14.972-17.390)	<0.001	1.318 (1.183-1.453)

Model 1, adjusted for age and gender; Model 2, adjusted for age, gender, coronary heart disease, hypertension, heart failure, diabetic retinopathy, hyperlipidemia,atherosclerosis, stroke, carotid occlusion, and arrhythmias; Model 3, adjusted for variables included in Model 2 and fatty liver, liver cirrhosis, other chronic liver diseases, biliary diseases, pancreatic diseases, renal impairment, gastrointestinal tumors, hematologic disorders, body mass index, glycosylated hemoglobin A1c, fasting blood glucose, triglyceride, high‐density lipoprotein cholesterol and hemoglobin.

OR, odds ratio; CI, confidence interval; SD, standard deviation.

### Subgroup analysis

**Figure 4:**
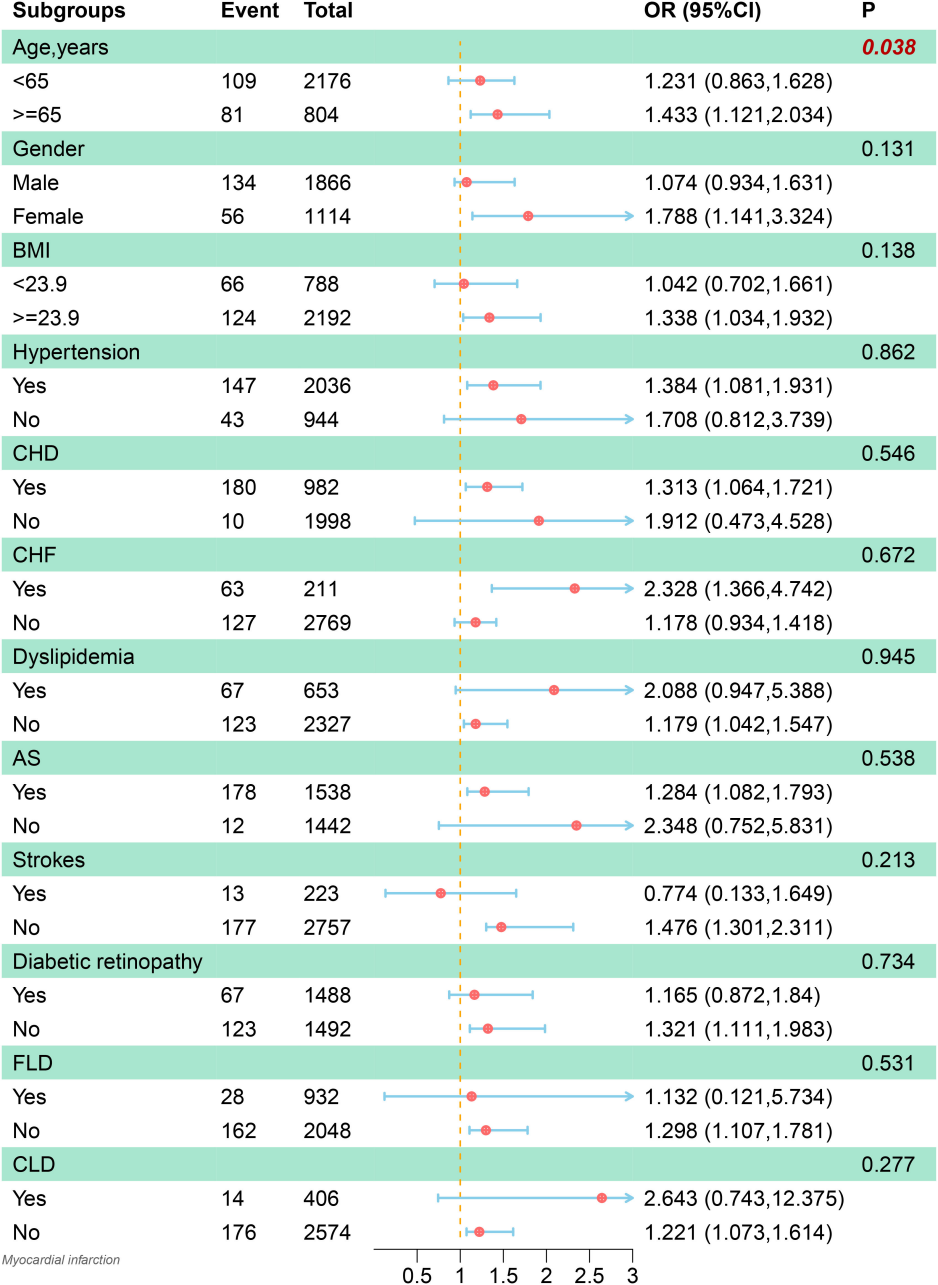
Subgroup analysis of the association between the Fibrosis-4 index and the incidence of myocardial infarction. The model was adjusted for age, gender, coronary heart disease, hypertension, heart failure, diabetic retinopathy, hyperlipidemia,atherosclerosis, stroke, carotid occlusion, arrhythmias, fatty liver, liver cirrhosis, other chronic liver diseases, biliary diseases, pancreatic diseases, renal impairment, gastrointestinal tumors, hematologic disorders, body mass index, glycosylated hemoglobin A1c, fasting blood glucose, triglyceride, high‐density lipoprotein cholesterol and hemoglobin. BMI, body mass index; CHD, coronary heart disease; CHF, chronic heart failure; AS, arteriosclerosis; FLD, fatty liver disease; CLD, other chronic liver diseases; OR, odds ratio; CI, confidence interval.

Detailed subgroup analyses were conducted to further explore the association between the FIB-4 and the co-occurrence of MI in patients with T2DM. The relationship between FIB-4 and the co-occurrence of MI was more significant in specific subgroups, as illustrated in **Figure 4**. These subgroups included the elderly, females, people with high BMI, hypertension, CHD, HF, and AS. Furthermore, this relationship was also significant in patients without hyperlipidemia, stroke, diabetic retinopathy, fatty liver disease, and other chronic liver diseases. Importantly, an interaction between age and the co-occurrence of MI was also observed (*p* = 0.02).

### Sensitivity analysis

Consistent findings were obtained across various methods to assess the primary results’ robustness. Specifically, the fully adjusted models applied to five imputed datasets confirmed the consistency of the primary analysis. Compared to Cluster 1, higher levels of FIB-4 (Clusters 3 and 4) were independently associated with the co-occurrence of MI in T2DM patients (OR, 95% CI: 1.948, 1.378 - 2.616, Cluster 3; OR, 95% CI: 16.132, 14.377 - 18.104, Cluster 4) (**Supplementary Table S6**). Results classified by FIB-4 risk thresholds and quintiles were consistent with the main study findings (OR, 95% CI: 3.140, 2.502 - 3.378, high-risk group; OR, 95% CI: 1.570, 1.084 - 1.956, quintile five groups) (**Supplementary Tables S7**, **S8**). To reduce selection bias resulting from non-random assignment, 1:1 propensity score matching was used, resulting in 181 similarly matched individuals with comparable baseline characteristics (**Supplementary Table S9**). The results continued to indicate a robust correlation between elevated FIB-4 levels and the co-occurrence of MI in T2DM patients (OR, 95% CI: 2.419, 1.562-3.276, Cluster 3; OR, 95% CI: 3.177, 2.918-3.436, Cluster 4; OR, 95% CI: 1.570, 1.265-1.857, per SD increase) (**Supplementary Table S10**). The results were further verified in a second independent dataset to minimize potential biases from a single center. The association between FIB-4 was a continuous variable despite the slightly reduced sample size (**Supplementary Table S11**). After adjusting for potential confounders, the results reaffirmed the independent association between FIB-4 and coexisting MI (OR, 95% CI 1.758, 1.144-2.702) (**Supplementary Table S12**). The ROC curve analysis of FIB-4 for MI demonstrated an area under the curve (AUC) of 0.562 (95% CI: 0.503–0.622), indicating a relatively weak ability of FIB-4 alone to distinguish MI. Based on the maximum Youden index (0.127), the optimal cutoff value was determined to be 1.45, with a sensitivity of 42.5% and a specificity of 70.2%.

## Discussion

This study collected cross-sectional data from two hospitals in China to investigate and validate the correlation between FIB-4 and the co-occurrence of MI in T2DM patients. The findings indicated that the elevated level of FIB-4 was independently correlated with the comorbidity of MI. However, no significant association was found with the co-occurrence of CHD. Fully adjusted RCS regression analysis demonstrated a linear relationship between FIB-4 levels and the co-occurrence of MI in patients with T2DM. Based on subgroup analyses, this association was more significant in females, adults over 65, those with a high BMI, and patients with hypertension, CHD, HF, and AS.

Liver fibrosis was initially evaluated using the FIB-4 index. In patients with T2DM, liver fibrosis is frequently concealed and prevalent (30). The significance of FIB-4 in CVD and metabolic disorders has been gaining attention due to the relationship of liver fibrosis with immune responses and inflammation, critical pathophysiological responses in T2DM, AS, and ischemic heart disease (31–33). It has been suggested that T2DM might act as a bridge correlating non-alcoholic fatty liver disease (NAFLD)/non-alcoholic steatohepatitis (NASH) with CVD (34). A recent large cohort study that followed obese and/or T2DM patients >10 years (n = 44,481) revealed that an increase in baseline FIB-4 levels was associated with a high risk of cardiovascular events (HR, 95% CI: 1.34, 1.21 to 1.48) and all-cause mortality (HR, 95% CI: 1.56, 1.45–1.68) after adjusting for age and gender (35). In coronary artery disease (CAD) in the Chinese population, cohort studies have observed a correlation between elevated levels of FIB-4 and an increase in all-cause (HR, 95% CI: 2.84, 2.14–3.76) and cardiovascular (HR, 95% CI: 3.34, 2.29–4.86) mortalities (21). Another multicenter cohort study on patients with stable CAD post-percutaneous coronary intervention showed that higher baseline FIB-4 levels (> 2.67) were substantially related to major adverse cardiac events, such as cardiovascular death, non-fatal MI, and ischemic stroke (HR, 1.17–1.77) (36), underscoring the importance of FIB-4 in predicting adverse outcomes in CHD patients.

The American Diabetes Association (ADA) recommends FIB-4 thresholds for assessing the risk of liver fibrosis (low risk: < 1.3; intermediate risk: 1.3-2.67; high risk: > 2.67) (18). However, considering the ethnic differences, K-means clustering was used to categorize the FIB-4 index. According to the current findings, clusters 3 and 4 are associated with a higher risk of co-occurring MI, as evidenced by FIB-4 levels > 2.41. The relatively lower FIB-4 threshold observed in this study is possibly due to racial specificity and disparities in disease pathophysiology. Despite limited research on the correlation between various FIB-4 risk thresholds and CVD events in T2DM patients, previous studies have indicated that FIB-4 is not successful in predicting liver fibrosis in non-Hispanic Black patients (37), suggesting that race plays a significant role in influencing the performance of FIB-4. Moreover, Kayadibi et al. (38) observed that changes in FIB-4 values are more significantly influenced by differences in PLT counts, suggesting that distinct risk thresholds could be determined for different diseases. Disease variations may also contribute to the differences in K-means clustering results, as this study focused on T2DM patients. Similar results were obtained via sensitivity analyses that employed these risk thresholds, indicating that this risk classification was appropriate for evaluating the risk in Chinese T2DM patients. Compared to previous studies, comprehensive adjustments for factors potentially influencing FIB-4 include coexisting conditions such as fatty liver disease, liver cirrhosis, chronic liver diseases, biliary diseases, and gastrointestinal tumors. The current results demonstrated a significant association between higher FIB-4 levels and the co-occurrence of MI in T2DM patients. However, the results were not statistically significant when evaluating this association with CHD, which is in line with the findings of studies conducted by Delgado (39) and Guan (23). A potential explanation for this discrepancy is that the definition of CHD in this study does not clearly distinguish between its subtypes. This also implies that the relationship between FIB-4 and co-occurring MI may be influenced by factors other than AS.

The results of the subgroup analysis indicate that the association between FIB-4 and MI is more significant in older individuals, those with a high BMI, and those with hypertension, CHD, CHF, or AS. These factors are directly associated with a higher risk of CVDs (25). A high sensitivity of FIB-4 may also be attributed to endothelial damage and inflammatory responses in the pathophysiological mechanisms (40). Interestingly, a significant correlation was also found among females and those without fatty liver disease or other chronic liver conditions. Postmenopausal women are more prone to metabolic syndrome and endothelial dysfunction, with decreased estrogen potentially leading to altered fat distribution and increased inflammation, thereby strengthening the association between FIB-4 and MI (41). In patients with chronic liver diseases, FIB-4 levels are commonly elevated as a result of the disease. In patients who do not have fatty liver disease or other chronic liver conditions, higher FIB-4 levels might more accurately indicate a relationship with CVD. These findings suggest that FIB-4 could potentially have significant clinical relevance in these groups. The interaction analysis revealed a statistically significant interaction between FIB-4 and age. However, since age is already incorporated into the calculation formula of FIB-4, this finding may partially reflect the inherent mathematical relationship within the formula rather than an independent biological effect, thus limiting its interpretative significance.

A multi-level sensitivity analysis was also carried out to improve the robustness of the current findings and minimize regression analysis errors due to the low number of positive cases in specific clusters. These analyses were consistent with the primary analysis. These findings were further validated in an independent dataset. However, during data collection for the validation cohort, detailed information on comorbidities could not be obtained due to storage limitations. Variables (LVEF, NTpro-BNP, Hb, HbA1c, GLU, and Cr) were adjusted with comorbidity information to reduce these errors. The validation data were obtained from a cardiovascular specialty ward. Compared to the discovery cohort, these patients were relatively elderly, had an increased number of females, a high prevalence of CHD and MI, and NTpro-BNP levels. This indicates that the validation cohort showed more severe CVD. Interestingly, the OR value for FIB-4 was higher in the validation cohort (1.758 vs. 1.318). This result aligns with Guan et al. (23), suggesting that FIB-4 is a reliable indicator across patients with different severity of CVD. Moreover, FIB-4 may possess high predictive value for higher-risk populations, highlighting its clinical and broad applicability in high-risk patients. Based on the Youden index, the optimal balance is achieved when FIB-4 = 1.45. This cutoff value is lower than the risk threshold determined by K-means clustering, which may be influenced by uncorrected confounding factors in the ROC analysis. Given the relatively low AUC, it suggests that the standalone use of FIB-4 is limited. However, its role in a combined model warrants further investigation in future studies.

This study was based on the outcomes of previous research to strengthen the relationship between FIB-4 and co-occurring MI in T2DM patients. Moreover, this study has several strengths. First, the current data included comprehensive and detailed co-morbidity data, allowing for well-adjusted true associations between FIB-4 and outcomes. Further, a novel categorization of FIB-4 levels was implemented via K-means clustering, as there were no risk stratification thresholds that could be used to evaluate the severity of liver fibrosis in Chinese T2DM patients. These results were then compared to the current ADA-recommended risk thresholds. Current findings demonstrated that the ADA-recommended thresholds also apply to the FIB-4 risk stratification in the Chinese T2DM population. However, K-means clustering results suggest that the risk thresholds for FIB-4 associated with MI in Chinese T2DM patients might be lower (2.41 vs. 2.67), indicating a need for further research to validate these findings. Finally, comprehensive subgroup and sensitivity analyses enhanced the credibility of the association between FIB-4 levels and co-occurring MI in T2DM patients.

Despite these strengths, the study also has some limitations. Since it was based on a cross-sectional survey, the causal and dynamic relationships between FIB-4 and MI could not be delineated, necessitating further longitudinal study designs to explore and validate these associations more accurately. Moreover, the association between FIB-4 levels and co-occurring MI may be less stable due to the limited sample size and the few cases of co-occurring MI despite the various sensitivity analyses. Further, due to storage limitations of the database in this study, disease diagnoses relied on recorded diagnostic labels. Although clinicians made these diagnoses, the lack of detailed diagnostic criteria references partially reduces the transparency of the study. Further investigation of FIB-4 is also restricted by the inability to acquire specific MI phenotypes and data on the extent of coronary stenosis. The lack of data on smoking history, alcohol, and drug consumption may have potential confounding factors that were not accounted for in the study. Lastly, despite validating the current data with information from another center, the findings were based on data collected from only two hospitals in Beijing, which may limit their generalizability. This underscores the need for additional regional and multicenter research to confirm the current findings.

## Conclusions

In conclusion, the study highlighted that elevated FIB-4 levels were significantly associated with an increased risk of MI in Chinese T2DM patients, particularly among the elderly, females, and those with comorbid conditions like high BMI, hypertension, CHD, and HF. Therefore, FIB-4, readily accessible, can be used as a valuable clinical marker for identifying high-risk patients with cardiovascular complications and tailoring personalized treatment strategies.

## Data Availability

The original contributions presented in the study are included in the article/**Supplementary Material**. Further inquiries can be directed to the corresponding author/s.
