# Supplementary figures and images for: Cross-sectional study on the association between the fibrosis-4 index and co-occurring myocardial infarction in Chinese patients with type 2 diabetes mellitus
Source: Front Endocrinol (Lausanne). 2025 Mar 12;16:1551472. doi: 10.3389/fendo.2025.1551472 (PMC11936788; doi:10.3389/fendo.2025.1551472)

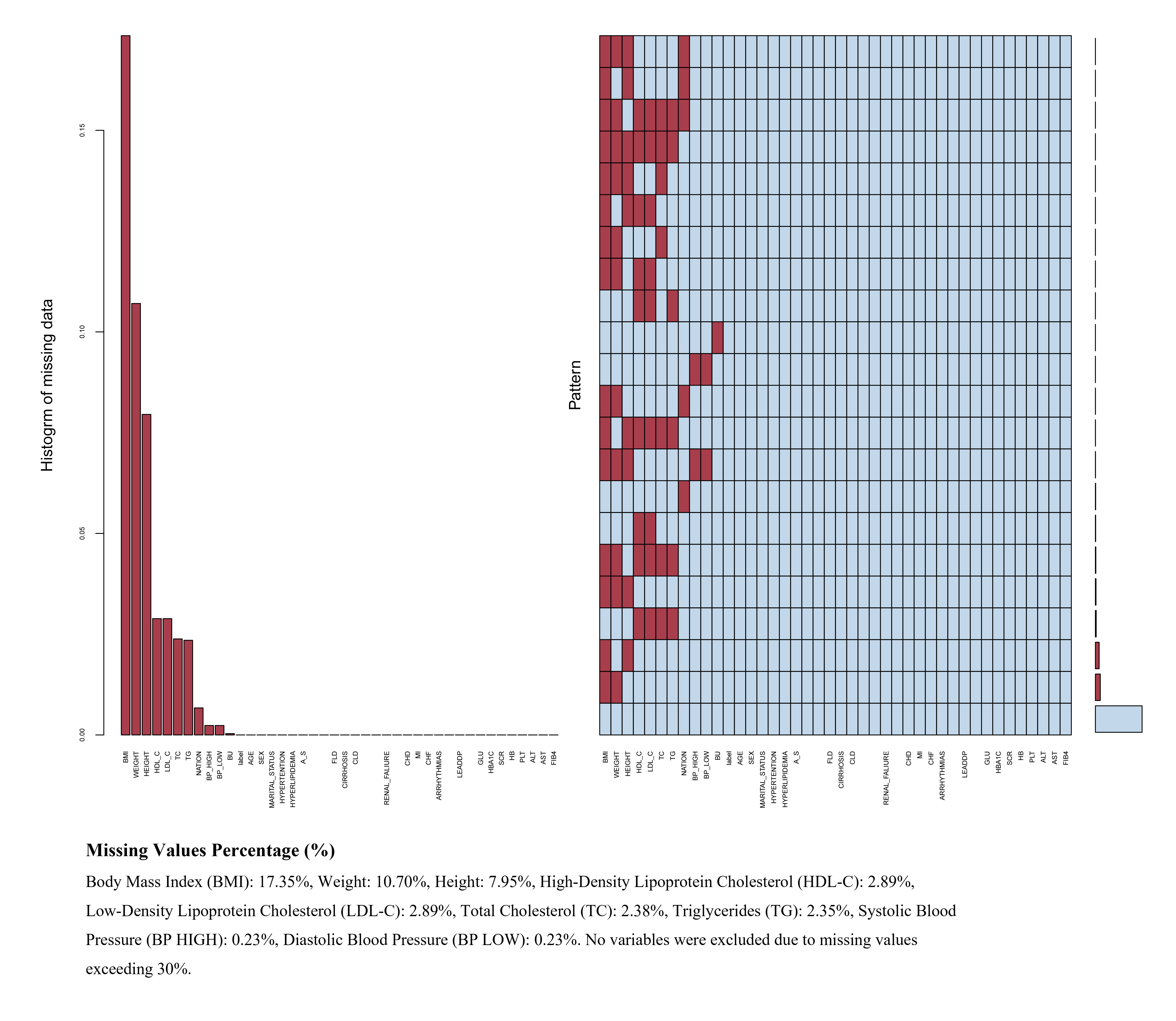

Supplement: Supplementary file 1 [file Image1.tif]
