# Supplementary material for: Cross-sectional study on the association between the fibrosis-4 index and co-occurring myocardial infarction in Chinese patients with type 2 diabetes mellitus
Source: Front Endocrinol (Lausanne). 2025 Mar 12;16:1551472. doi: 10.3389/fendo.2025.1551472 (PMC11936788; doi:10.3389/fendo.2025.1551472)
Supplement: Supplementary file 2 [file Table1.docx]

**Catalogs**

[Table S1. The examination of multicollinearity among covariates in the discovery phase 1](#_Toc15176)

[Table S2. Baseline characteristics of validation data 2](#_Toc22658)

[Table S3. Individual baseline characteristics by outcome before interpolation 3](#_Toc8692)

[Table S4. Individual baseline characteristics by outcome after interpolation 4](#_Toc7786)

[Table S5. Association between Fibrosis-4 index and coronary heart disease 5](#_Toc10773)

[Table S6. Association Between Fibrosis-4 Index and myocardial infarction in All Interpolated Datasets 6](#_Toc8609)

[Table S7. Association between the Fibrosis-4 index and myocardial infarction according to risk thresholds 6](#_Toc21061)

[Table S8. Association between the Fibrosis-4 index and myocardial infarction according to quintile classification 7](#_Toc1454)

[Table S9. Baseline characteristics of individuals by outcome after propensity score matching 8](#_Toc14822)

[Table S10. Association between Fibrosis-4 index and myocardial infarction after propensity score matching 9](#_Toc5784)

[Table S11. Tests for multicollinearity between covariates in the validation phase 10](#_Toc9824)

[Table S12. Association between Fibrosis-4 index and myocardial infarction in validation data 11](#_Toc4000)

# Table S1. The examination of multicollinearity among covariates in the discovery phase

| **Covariates** | **VIF** |
| --- | --- |
| TC | 14.30 |
| LDL-C | 10.61 |
| AST | 6.74 |
| ALT | 5.00 |
| Diabetic retinopathy | 4.03 |
| TG | 3.66 |
| FIB-4 | 3.54 |
| AS | 2.21 |
| CHD | 2.18 |
| Hb | 2.14 |
| HDL-C | 1.86 |
| PLT | 1.86 |
| SBP | 1.86 |
| DBP | 1.76 |
| Age | 1.72 |
| HbA1c | 1.54 |
| Sex | 1.48 |
| LEADDP | 1.37 |
| Diabetic retinopathy | 1.37 |
| FBG | 1.36 |
| FLD | 1.32 |
| Hypertension | 1.31 |
| Renal failure | 1.30 |
| Cirrhosis | 1.23 |
| BMI | 1.22 |
| CHF | 1.19 |
| Dyslipidemia | 1.19 |
| CLD | 1.16 |
| Digestive tumors | 1.12 |
| Stroke | 1.09 |
| Biliary tract diseases | 1.08 |
| Pancreatic Diseases | 1.05 |
| Carotid artery stenosis | 1.05 |
| Marital status | 1.04 |

Abbreviations: VIF, Variance Inflation Factor; TC, total cholesterol; LDL-C, low-density lipoprotein cholesterol; AST, aspartate aminotransferase; ALT, alanine aminotransferase; TG, triglycerides; FIB-4, Fibrosis-4 index; AS, atherosclerosis; CHD, coronary heart disease; Hb, hemoglobin; HDL-C, high-density lipoprotein cholesterol; PLT, platelets; SBP, systolic blood pressure; DBP, diastolic blood pressure; HbA1c, hemoglobin A1c; LEADDP, lower extremity artery disease; Diabetic retinopathy, diabetic retinopathy; FBG, fasting blood glucose; FLD, fatty liver disease; BMI, body mass index; CHF, chronic heart failure; CLD, chronic liver disease.

# Table S2. Baseline characteristics of validation data

| **Characteristics** | **Non - MI** | **MI** | **P-value** |
| --- | --- | --- | --- |
| N | 203 | 21 |  |
| Gender (Female) | 97 (47.8%) | 10 (47.6%) | 1.000 |
| Age, years | 66.8 ± 9.6 | 62.7 ± 12.7 | 0.163 |
| Smoking history | 73 (36.0%) | 9 (42.9%) | 0.669 |
| SBP, mmHg | 147.5 ± 19.7 | 139.2 ± 20.0 | 0.069 |
| DBP, mmHg | 82.3 ± 11.5 | 80.1 ± 9.7 | 0.391 |
| BMI, kg/m^2^ | 26.1 ± 4.1 | 26.3 ± 3.8 | 0.822 |
| CHD | 188 (92.6%) | 21 (100.0%) | 0.372 |
| Hypertension | 157 (77.3%) | 15 (71.4%) | 0.588 |
| ALT, U/L | 20.0 (14.0, 31.0) | 27.0 (16.0, 34.0) | 0.280 |
| AST, U/L | 18.0 (15.0, 22.0) | 20.0 (17.0, 30.0) | 0.068 |
| PLT, 10^^9^/L | 214.0 ± 54.3 | 216.0 ± 84.1 | 0.884 |
| FIB-4 | 1.3 (1.0, 1.6) | 1.3 (1.0, 1.8) | 0.026 |
| FBG, mmol/L | 7.8 ± 2.8 | 7.9 ± 2.8 | 0.869 |
| HbA1c, % | 7.6 ± 1.4 | 7.6 ± 1.8 | 0.992 |
| TC, mmol/L | 4.0 ± 1.2 | 4.1 ± 1.2 | 0.533 |
| TG, mmol/L | 1.4 (1.0, 1.8) | 1.7 (1.2, 2.2) | 0.118 |
| HDL-c, mmol/L | 1.1 ± 0.2 | 1.0 ± 0.2 | 0.042 |
| LDL-c, mmol/L | 2.4 ± 0.8 | 2.6 ± 0.9 | 0.304 |
| RBC, 10^^9^/L | 4.4 ± 0.7 | 4.3 ± 0.7 | 0.362 |
| Hb, g/L | 132.9 ± 18.2 | 127.8 ± 20.6 | 0.229 |
| BUN, mmol/L | 5.7 (4.8, 7.0) | 5.1 (4.1, 7.1) | 0.506 |
| Cr, mmol/L | 67.3 (58.8, 81.1) | 68.5 (59.4, 91.7) | 0.374 |
| LVEF,% | 65.1 ± 7.8 | 59.9 ± 15.9 | 0.010 |
| NTpro-BNP, pg/ml | 116.0 (57.0, 260.5) | 408.0 (134.0, 1913.0) | <0.001 |

Note: Variables are expressed as mean ± standard deviation or quartiles (M25, M75) or n (%).

Abbreviations: SBP, systolic blood pressure; DBP, diastolic blood pressure; BMI, body mass index; CHD, coronary heart disease; ALT, alanine aminotransferase; AST, aspartate aminotransferase; PLT, platelet count; FIB-4, Fibrosis-4 index; FBG, fasting blood glucose; HbA1c, glycosylated hemoglobin A1c; TC, total cholesterol; TG, triglyceride; HDL‐c, high‐density lipoprotein cholesterol; LDL-c, low-density lipoprotein cholesterol; RBC, red blood cell count; Hb, hemoglobin; BUN, blood urea nitrogen; Cr, creatinine; LVEF, left ventricular ejection fraction; NTpro-BNP, N-terminal pro b-type natriuretic peptide.

# Table S3. Individual baseline characteristics by outcome before interpolation

| **Characteristics** | **Non - MI** | **MI** | **P-value** |
| --- | --- | --- | --- |
| N | 2790 | 190 |  |
| Gender (Female) | 1058 (37.9%) | 56 (29.5%) | 0.024 |
| Age, years | 57.4 ± 11.1 | 63.3 ± 11.0 | <0.001 |
| Marital status (Married) | 2734 (98.0%) | 183 (96.3%) | 0.118 |
| SBP, mmHg | 139.1 ± 21.1 | 132.7 ± 18.9 | <0.001 |
| DBP, mmHg | 80.8 ± 12.0 | 76.9 ± 11.3 | <0.001 |
| BMI, kg/m^2^ | 26.4 ± 3.8 | 25.8 ± 3.9 | 0.084 |
| CHD | 802 (28.8%) | 180 (94.7%) | <0.001 |
| Hypertension | 1889 (67.7%) | 147 (77.4%) | 0.007 |
| Dyslipidemia | 586 (21.0%) | 67 (35.3%) | <0.001 |
| Diabetic retinopathy | 1421 (50.9%) | 67 (35.3%) | <0.001 |
| Arteriosclerosis | 1360 (48.8%) | 178 (93.7%) | <0.001 |
| CHF | 148 (5.3%) | 63 (33.2%) | <0.001 |
| Stroke | 210 (7.5%) | 13 (6.8%) | 0.838 |
| Carotid artery stenosis | 120 (4.3%) | 9 (4.7%) | 0.919 |
| Fatty liver disease | 904 (32.4%) | 28 (14.7%) | <0.001 |
| Other chronic liver diseases | 392 (14.1%) | 14 (7.4%) | 0.013 |
| Cirrhosis | 46 (1.7%) | 1 (0.5%) | 0.365 |
| Pancreatic Diseases | 46 (1.7%) | 2 (1.1%) | 0.767 |
| Biliary tract diseases | 403 (14.4%) | 22 (11.6%) | 0.324 |
| Digestive tumors | 150 (5.4%) | 3 (1.6%) | 0.034 |
| Renal failure | 166 (6.0%) | 14 (7.4%) | 0.524 |
| LEAD | 444 (15.9%) | 29 (15.3%) | 0.893 |
| ALT, U/L | 18.0 (12.7, 27.1) | 20.9 (13.5, 31.8) | 0.011 |
| AST, U/L | 16.3 (13.1, 21.1) | 18.2 (14.0, 27.2) | <0.001 |
| PLT, 10^^9^/L | 218.5 ± 71.5 | 218.0 ± 74.5 | 0.931 |
| Hb, g/L | 131.71 ± 23.21 | 132.38 ± 21.57 | 0.698 |
| FIB-4 | 1.1 (0.8, 1.4) | 1.3 (0.9, 2.0) | <0.001 |
| FBG, mmol/L | 8.4 ± 3.9 | 9.5 ± 4.2 | <0.001 |
| HbA1c, % | 7.8 ± 1.8 | 7.8 ± 1.7 | 0.775 |
| TC, mmol/L | 4.5 (3.8, 5.3) | 4.0 (3.3, 4.9) | <0.001 |
| TG, mmol/L | 1.6 (1.1, 2.3) | 1.6 (1.1, 2.2) | 0.800 |
| HDL-c, mmol/L | 1.1 ± 0.3 | 1.0 ± 0.2 | <0.001 |
| LDL-c, mmol/L | 2.9 ± 1.2 | 2.5 ± 1.0 | <0.001 |

Note: Variables are expressed as mean ± standard deviation or quartiles (M25, M75) or n (%).

Abbreviations: SBP, systolic blood pressure; DBP, diastolic blood pressure; BMI, body mass index; CHD, coronary heart disease; CHF, chronic heart failure. LEAD, Atherosclerotic lesions of the lower limbs; ALT, alanine aminotransferase; AST, aspartate aminotransferase; PLT, platelet count; Hb, hemoglobin; FIB-4, Fibrosis-4 index; FBG, fasting blood glucose; HbA1c, glycosylated hemoglobin A1c; TC, total cholesterol; TG, triglyceride; HDL‐c, high‐density lipoprotein cholesterol; LDL-c, low-density lipoprotein cholesterol.

# Table S4. Individual baseline characteristics by outcome after interpolation

| **Characteristics** | **Non - MI** | **MI** | **P-value** |
| --- | --- | --- | --- |
| N | 2790 | 190 |  |
| Gender (Female) | 1058 (37.9%) | 56 (29.5%) | 0.024 |
| Age, years | 57.4 ± 11.1 | 63.3 ± 11.0 | <0.001 |
| Marital status (Married) | 2734 (98.0%) | 183 (96.3%) | 0.118 |
| SBP, mmHg | 139.1 ± 21.1 | 132.7 ± 18.9 | <0.001 |
| DBP, mmHg | 80.8 ± 12.0 | 76.9 ± 11.3 | <0.001 |
| BMI, kg/m^2^ | 26.4 ± 3.8 | 25.8 ± 3.9 | 0.084 |
| CHD | 802 (28.8%) | 180 (94.7%) | <0.001 |
| Hypertension | 1889 (67.7%) | 147 (77.4%) | 0.007 |
| Dyslipidemia | 586 (21.0%) | 67 (35.3%) | <0.001 |
| Diabetic retinopathy | 1421 (50.9%) | 67 (35.3%) | <0.001 |
| Arteriosclerosis | 1360 (48.8%) | 178 (93.7%) | <0.001 |
| CHF | 148 (5.3%) | 63 (33.2%) | <0.001 |
| Stroke | 210 (7.5%) | 13 (6.8%) | 0.838 |
| Carotid artery stenosis | 120 (4.3%) | 9 (4.7%) | 0.919 |
| Fatty liver disease | 904 (32.4%) | 28 (14.7%) | <0.001 |
| Other chronic liver diseases | 392 (14.1%) | 14 (7.4%) | 0.013 |
| Cirrhosis | 46 (1.7%) | 1 (0.5%) | 0.365 |
| Pancreatic Diseases | 46 (1.7%) | 2 (1.1%) | 0.767 |
| Biliary tract diseases | 403 (14.4%) | 22 (11.6%) | 0.324 |
| Digestive tumors | 150 (5.4%) | 3 (1.6%) | 0.034 |
| Renal failure | 166 (6.0%) | 14 (7.4%) | 0.524 |
| LEAD | 444 (15.9%) | 29 (15.3%) | 0.893 |
| ALT, U/L | 18.0 (12.7, 27.1) | 20.9 (13.5, 31.8) | 0.011 |
| AST, U/L | 16.3 (13.1, 21.1) | 18.2 (14.0, 27.2) | <0.001 |
| PLT, 10^^9^/L | 218.5 ± 71.5 | 218.0 ± 74.5 | 0.931 |
| Hb, g/L | 131.34 ± 22.78 | 132.67 ± 21.32 | 0.702 |
| FIB-4 | 1.1 (0.8, 1.4) | 1.3 (0.9, 2.0) | <0.001 |
| FBG, mmol/L | 8.4 ± 3.9 | 9.5 ± 4.2 | <0.001 |
| HbA1c, % | 7.8 ± 1.8 | 7.8 ± 1.7 | 0.775 |
| TC, mmol/L | 4.5 (3.8, 5.3) | 4.0 (3.3, 4.9) | <0.001 |
| TG, mmol/L | 1.6 (1.1, 2.3) | 1.6 (1.1, 2.2) | 0.800 |
| HDL-c, mmol/L | 1.1 ± 0.3 | 1.0 ± 0.2 | <0.001 |
| LDL-c, mmol/L | 2.9 ± 1.2 | 2.5 ± 1.0 | <0.001 |

Note: Variables are expressed as mean ± standard deviation or quartiles (M25, M75) or n (%).

Abbreviations: SBP, systolic blood pressure; DBP, diastolic blood pressure; BMI, body mass index; CHD, coronary heart disease; CHF, chronic heart failure. LEAD, Atherosclerotic lesions of the lower limbs; ALT, alanine aminotransferase; AST, aspartate aminotransferase; PLT, platelet count; Hb, hemoglobin; FIB-4, Fibrosis-4 index; FBG, fasting blood glucose; HbA1c, glycosylated hemoglobin A1c; TC, total cholesterol; TG, triglyceride; HDL‐c, high‐density lipoprotein cholesterol; LDL-c, low-density lipoprotein cholesterol.

#

# Table S5. Association between Fibrosis-4 index and coronary heart disease

| **Fibrosis-4 index** | **Cluster** | | | | | **Continuous** |
| --- | --- | --- | --- | --- | --- | --- |
|  | **Cluster 1** | **Cluster 2** | **Cluster 3** | **Cluster 4** | **P for trend** | **Per 1 SD increase** |
| Median | 0.84 | 1.51 | 2.95 | 7.12 |  |  |
| Cases, n (%) | 536 (29.4%) | 359 (37.6%) | 75 (43.9%) | 12 (44.4%) | - |  |
| Crude, OR (95%CI) | Reference | 1.447 (1.227-1.707) | 1.880 (1.368-2.585) | 1.925 (0.852-4.141) | <0.001 | 1.256 (1.156-1.364) |
| Model 1, OR (95%CI) | Reference | 0.807 (0.670-0.972) | 0.865 (0.610-1.227) | 1.270 (0.652-2.871) | 0.713 | 1.007 (0.923-1.099) |
| Model 2, OR (95%CI) | Reference | 0.794 (0.619-1.020) | 1.023 (0.611-1.715) | 1.332 (0.422-4.205) | 0.931 | 1.044 (0.927-1.176) |
| Model 3, OR (95%CI) | Reference | 0.816 (0.576-1.057) | 0.807 (0.319-1.294) | 0.644 (-0.631-1.919) | 0.188 | 0.967 (0.862-1.073) |

Note: Model 1, adjusted for age and gender; Model 2, adjusted for age, gender, myocardial infarction, hypertension, heart failure, diabetic retinopathy, hyperlipidemia,atherosclerosis, stroke, carotid occlusion, and arrhythmias; Model 3, adjusted for variables included in Model 2 and fatty liver, liver cirrhosis, other chronic liver diseases, biliary diseases, pancreatic diseases, renal impairment, gastrointestinal tumors, hematologic disorders, body mass index, glycosylated hemoglobin A1c, fasting blood glucose, triglyceride, high‐density lipoprotein cholesterol and hemoglobin.

Abbreviations: OR, odds ratio; CI, confidence interval; SD, standard deviation.

# Table S6. Association Between Fibrosis-4 Index and myocardial infarction in All Interpolated Datasets

| **Fibrosis-4 index** | **Cluster** | | | |
| --- | --- | --- | --- | --- |
|  | **Cluster 1** | **Cluster 2** | **Cluster 3** | **Cluster 4** |
| Cases, n (%) | 91 (5.0) | 63 (6.6) | 26 (15.2) | 10 (37.0) |
| Crude, OR (95%CI) | Reference | 1.345 (0.966-1.873) | 3.419 (2.142-5.457) | 11.215 (4.994-25.187) |
| Model 1, OR (95%CI) | Reference | 0.861 (0.601-1.232) | 1.872 (1.124-3.119) | 8.766 (3.779-20.333) |
| Model 2, OR (95%CI) | Reference | 0.931 (0.631-1.373) | 1.969 (1.101-3.522) | 13.279 (4.285-41.154) |
| Model 3, OR (95%CI) | Reference | 0.966 (0.521-1.399) | 1.948 (1.378-2.616) | 16.132 (14.377-18.104) |

Note: Model 1, adjusted for age and gender; Model 2, adjusted for age, gender, coronary heart disease, hypertension, heart failure, diabetic retinopathy, hyperlipidemia,atherosclerosis, stroke, carotid occlusion, and arrhythmias; Model 3, adjusted for variables included in Model 2 and fatty liver, liver cirrhosis, other chronic liver diseases, biliary diseases, pancreatic diseases, renal impairment, gastrointestinal tumors, hematologic disorders, body mass index, glycosylated hemoglobin A1c, fasting blood glucose, triglyceride, high‐density lipoprotein cholesterol and hemoglobin.

Abbreviations: OR, odds ratio; CI, confidence interval; SD, standard deviation

# Table S7. Association between the Fibrosis-4 index and myocardial infarction according to risk thresholds

| **Fibrosis-4 index** | **Risk** | | | |
| --- | --- | --- | --- | --- |
|  | **Low risk** | **Intermediate risk** | **High risk** | **P for trend** |
| Median | 0.86 | 1.62 | 3.48 | - |
| Cases, n (%) | 96 (4.8) | 67 (7.9) | 27 (18.6) | - |
| Crude, OR (95%CI) | Reference | 1.684 (1.219-2.326) | 4.502 (2.826-7.173) | <0.001 |
| Model 1, OR (95%CI) | Reference | 1.123 (0.791-1.596) | 2.766 (1.677-4.563) | <0.001 |
| Model 2, OR (95%CI) | Reference | 1.205 (0.822-1.765) | 2.908 (1.612-5.244) | <0.001 |
| Model 3, OR (95%CI) | Reference | 1.303 (0.910-1.697) | 3.140 (2.502-3.778) | 0.017 |

Note: Model 1, adjusted for age and gender; Model 2, adjusted for age, gender, coronary heart disease, hypertension, heart failure, diabetic retinopathy, hyperlipidemia,atherosclerosis, stroke, carotid occlusion, and arrhythmias; Model 3, adjusted for variables included in Model 2 and fatty liver, liver cirrhosis, other chronic liver diseases, biliary diseases, pancreatic diseases, renal impairment, gastrointestinal tumors, hematologic disorders, body mass index, glycosylated hemoglobin A1c, fasting blood glucose, triglyceride, high‐density lipoprotein cholesterol and hemoglobin.

Abbreviations: OR, odds ratio; CI, confidence interval; SD, standard deviation.

# Table S8. Association between the Fibrosis-4 index and myocardial infarction according to quintile classification

| **Fibrosis-4 index** | **Quintiles** | | | | | |
| --- | --- | --- | --- | --- | --- | --- |
|  | **Quintile 1** | **Quintile 2** | **Quintile 3** | **Quintile 4** | **Quintile 5** | **P for trend** |
| Median | 0.58 | 0.83 | 1.06 | 1.36 | 2.08 | - |
| Cases, n (%) | 29 (4.9) | 25 (4.2) | 35 (5.9) | 30 (5.0) | 71 (12.0) | - |
| Crude, OR (95%CI) | Reference | 0.856 (0.495-1.480) | 1.220 (0.736-2.023) | 1.036 (0.614-1.749) | 2.644 (1.689-4.138) | <0.001 |
| Model 1, OR (95%CI) | Reference | 0.842 (0.487-1.456) | 1.229 (0.741-2.039) | 1.403 (0.618-1.762) | 2.683 (1.713-4.203) | 0.046 |
| Model 2, OR (95%CI) | Reference | 0.622 (0.349-1.106) | 0.936 (0.549-1.598) | 0.728 (0.419-1.266) | 1.747 (1.078-2.833) | 0.005 |
| Model 3, OR (95%CI) | Reference | 0.606 (-0.015-1.226) | 0.876 (0.277-1.474) | 0.665 (0.043-1.288) | 1.570 (1.084-1.956) | 0.042 |

Note: Model 1, adjusted for age and gender; Model 2, adjusted for age, gender, coronary heart disease, hypertension, heart failure, diabetic retinopathy, hyperlipidemia,atherosclerosis, stroke, carotid occlusion, and arrhythmias; Model 3, adjusted for variables included in Model 2 and fatty liver, liver cirrhosis, other chronic liver diseases, biliary diseases, pancreatic diseases, renal impairment, gastrointestinal tumors, hematologic disorders, body mass index, glycosylated hemoglobin A1c, fasting blood glucose, triglyceride, high‐density lipoprotein cholesterol and hemoglobin.

Abbreviations: OR, odds ratio; CI, confidence interval; SD, standard deviation

# Table S9. Baseline characteristics of individuals by outcome after propensity score matching

| **Characteristics** | **Non - MI** | **MI** | **P-value** |
| --- | --- | --- | --- |
| N | 181 | 181 |  |
| Gender (Female) | 57 (31.5%) | 55 (30.4%) | 0.909 |
| Age, years | 62.3 ± 11.0 | 63.7 ± 10.8 | 0.231 |
| SBP, mmHg | 140.4 ± 20.7 | 138.4 ± 17.3 | 0.724 |
| DBP, mmHg | 78.3 ± 12.9 | 76.8 ± 11.3 | 0.284 |
| BMI, kg/m^2^ | 27.2 ± 4.0 | 27.1 ± 5.0 | 0.745 |
| CHD | 171 (94.5%) | 171 (94.5%) | 1.000 |
| Hypertension | 142 (78.5%) | 139 (76.8%) | 0.801 |
| Dyslipidemia | 63 (34.8%) | 65 (35.9%) | 0.912 |
| Diabetic retinopathy | 75 (41.4%) | 65 (35.9%) | 0.331 |
| Arteriosclerosis | 167 (92.3%) | 169 (93.4%) | 0.839 |
| CHF | 53 (29.3%) | 54 (29.8%) | 1.000 |
| Stroke | 21 (11.6%) | 14 (7.7%) | 0.336 |
| Carotid artery stenosis | 11 (6.1%) | 8 (4.4%) | 0.637 |
| Fatty liver disease | 42 (23.2%) | 27 (14.9%) | 0.061 |
| Other chronic liver diseases | 19 (10.5%) | 13 (7.2%) | 0.355 |
| Cirrhosis | 4 (2.2%) | 1 (0.6%) | 0.368 |
| Pancreatic Diseases | 0 (0.0%) | 2 (1.1%) | 0.478 |
| Biliary tract diseases | 24 (13.3%) | 21 (11.6%) | 0.750 |
| Digestive tumors | 8 (4.4%) | 3 (1.7%) | 0.221 |
| Renal failure | 17 (9.4%) | 11 (6.1%) | 0.325 |
| ALT, U/L | 18.7 (12.9, 30.1) | 20.9 (13.3, 32.0) | 0.319 |
| AST, U/L | 16.8 (12.6, 23.1) | 18.2 (14.1, 27.4) | 0.013 |
| PLT, 10^^9^/L | 213.3 ± 71.8 | 215.4 ± 69.2 | 0.773 |
| Hb, g/L | 129.59 ± 23.5 | 133.03 ± 20.7 | 0.140 |
| FIB-4 | 1.2 (0.8, 1.7) | 1.3 (0.9, 2.0) | 0.040 |
| FBG, mmol/L | 9.8 ± 4.5 | 9.2 ± 4.0 | 0.188 |
| HbA1c, % | 8.0 ± 1.7 | 7.8 ± 1.6 | 0.255 |
| TC, mmol/L | 4.2 (3.4, 5.0) | 4.1 (3.4, 4.9) | 0.472 |
| TG, mmol/L | 1.6 (1.0, 2.4) | 1.6 (1.1, 2.2) | 0.951 |
| HDL-c, mmol/L | 1.0 ± 0.3 | 1.0 ± 0.2 | 0.726 |
| LDL-c, mmol/L | 2.6 ± 1.0 | 2.5 ± 1.0 | 0.655 |

Note: Variables are expressed as mean ± standard deviation or quartiles (M25, M75) or n (%).

Abbreviations: SBP, systolic blood pressure; DBP, diastolic blood pressure; BMI, body mass index; CHD, coronary heart disease; CHF, chronic heart failure. LEAD, Atherosclerotic lesions of the lower limbs; ALT, alanine aminotransferase; AST, aspartate aminotransferase; PLT, platelet count; Hb, hemoglobin; FIB-4, Fibrosis-4 index; FBG, fasting blood glucose; HbA1c, glycosylated hemoglobin A1c; TC, total cholesterol; TG, triglyceride; HDL‐c, high‐density lipoprotein cholesterol; LDL-c, low-density lipoprotein cholesterol.

# Table S10. Association between Fibrosis-4 index and myocardial infarction after propensity score matching

| **Fibrosis-4 index** | **Cluster** | | | | | **Continuous** |
| --- | --- | --- | --- | --- | --- | --- |
|  | **Cluster 1** | **Cluster 2** | **Cluster 3** | **Cluster 4** | **P for trend** | **Per 1 SD increase** |
| Median | 0.86 | 1.63 | 2.91 | 7.21 | - |  |
| Cases, n (%) | 86 (46.7) | 61 (48.0) | 24 (60.0) | 10 (90.9) | - |  |
| Crude, OR (95%CI) | Reference | 1.053 (0.600-1.506) | 1.709 (1.014-2.405) | 11.395 (9.320-13.471) | 0.004 | 1.368 (1.114-1.622) |
| Model 1, OR (95%CI) | Reference | 0.982 (0.499-1.466) | 1.543 (0.807-2.279) | 10.995 (8.915-13.076) | 0.007 | 1.338 (1.080-1.595) |
| Model 2, OR (95%CI) | Reference | 0.955 (0.464-1.446) | 1.459 (0.707-2.212) | 11.153 (9.052-13.254) | 0.009 | 1.322 (1.068-1.576) |
| Model 3, OR (95%CI) | Reference | 0.975 (0.454-1.495) | 2.419 (1.562-3.276) | 3.177 (2.918-3.436) | 0.001 | 1.570 (1.265-1.875) |

Note: Model 1, adjusted for age and gender; Model 2, adjusted for age, gender, coronary heart disease, hypertension, heart failure, diabetic retinopathy, hyperlipidemia,atherosclerosis, stroke, carotid occlusion, and arrhythmias; Model 3, adjusted for variables included in Model 2 and fatty liver, liver cirrhosis, other chronic liver diseases, biliary diseases, pancreatic diseases, renal impairment, gastrointestinal tumors, hematologic disorders, body mass index, glycosylated hemoglobin A1c, fasting blood glucose, triglyceride, high‐density lipoprotein cholesterol and hemoglobin.

Propensity score matching was performed using the nearest-neighbor matching method, with caliper matching applied to enhance precision. The caliper width was set at 0.2 to further improve matching accuracy. The selection of matching variables was based on factors independently associated with myocardial infarction in logistic regression analysis, combined with clinical expertise and previous literature. Ultimately, sex, hypertension, hyperlipidemia, coronary artery disease, chronic heart failure, body mass index, fasting blood glucose, glycated hemoglobin, and high-density lipoprotein cholesterol levels were chosen as matching variables. Given the high collinearity between total cholesterol and low-density lipoprotein cholesterol with myocardial infarction, these two variables were not separately adjusted to avoid multicollinearity and excessive adjustment.

Abbreviations: OR, odds ratio; CI, confidence interval; SD, standard deviation.

# Table S11. Tests for multicollinearity between covariates in the validation phase

| **Covariates** | **VIF** |
| --- | --- |
| AST | 10.66 |
| TC | 7.84 |
| LDL-C | 6.74 |
| Hb | 6.09 |
| RBC | 5.73 |
| FIB-4 | 5.72 |
| ALT | 4.30 |
| Gender | 2.91 |
| PLT | 2.78 |
| DBP | 2.50 |
| SBP | 2.44 |
| HDL-C | 2.08 |
| Smoking history | 2.03 |
| Cr | 1.98 |
| FBG | 1.82 |
| HbA1c | 1.82 |
| Age | 1.80 |
| NTpro-BNP | 1.66 |
| LVEF | 1.59 |
| TG | 1.45 |
| Hypertension | 1.28 |
| BMI | 1.26 |
| CHD | 1.21 |

Abbreviations: VIF, Variance Inflation Factor; AST, aspartate aminotransferase; TC, total cholesterol; LDL-C, low-density lipoprotein cholesterol; Hb, hemoglobin; RBC, red blood cells; FIB-4, Fibrosis-4 index; ALT, alanine aminotransferase; PLT, platelets; DBP, diastolic blood pressure; SBP, systolic blood pressure; HDL-C, high-density lipoprotein cholesterol; Cr, creatinine; FBG, fasting blood glucose; HbA1c, hemoglobin A1c; NTpro-BNP, N-terminal pro b-type natriuretic peptide; LVEF, left ventricular ejection fraction; TG, triglycerides; BMI, body mass index; CHD, coronary heart disease.

#

# Table S12. Association between Fibrosis-4 index and myocardial infarction in validation data

| **Fibrosis-4 index** |  |
| --- | --- |
|  | **Per 1 SD increase** |
| Cases, n (%) | 21 (9.4) |
| Crude, OR (95%CI) | 1.495 (1.025-2.182) |
| Model 1, OR (95%CI) | 1.629 (1.115-2.381) |
| Model 2, OR (95%CI) | 1.758 (1.144-2.702) |

Note: Model 1, adjusted for age, gender and smoking history; Model 2, adjusted for age, gender, smoking history, body mass index, systolic blood pressure, diastolic blood pressure, hypertension history, left ventricular ejection fraction, N-terminal pro b-type natriuretic peptide, glycosylated hemoglobin A1c,fasting blood glucose and creatinine. Total cholesterol, low-density lipoprotein cholesterol, hemoglobin and red blood cells was excluded from the model because it had a VIF value greater than five. Since FIB-4 is the observed independent variable, and after removing variables with a VIF greater than five, the subsequent multicollinearity test showed a significant reduction in the VIF value of FIB-4 (1.888), it can be concluded that multicollinearity between the independent and dependent variables has been reduced.

Abbreviations: OR, odds ratio; CI, confidence interval; SD, standard deviation.
